# Emotionale Entwicklung bei Menschen mit Störungen der Intelligenzentwicklung: klinische und therapeutische Perspektiven

**DOI:** 10.1007/s00115-026-01978-z

**Published:** 2026-05-11

**Authors:** Tanja Sappok

**Affiliations:** Krankenhaus Mara gGmbH – Universitätsklinik für Inklusive Medizin, Medizin für Menschen mit Behinderungen, Universitätsklinikum OWL der Universität Bielefeld, Campus Bielefeld-Bethel, Maraweg 21, 33617 Bielefeld, Deutschland

**Keywords:** Emotionale Entwicklung, Psychische Gesundheit, Verhaltensstörungen, Diagnostik, Störungen der Intelligenzentwicklung, Emotional functioning, Mental health, Behavioral disorders, Diagnostics, Intellectual disability

## Abstract

**Hintergrund:**

Menschen mit Störungen der Intelligenzentwicklung (SIE) weisen neben kognitiven auch emotionale Entwicklungsverzögerungen auf, die mit herausforderndem Verhalten und eingeschränkter Teilhabe sowie häufig nicht notwendiger Psychopharmakotherapie einhergehen können. Der emotionale Entwicklungsansatz bietet einen Rahmen, entwicklungsassoziierte Verhaltensweisen zu verstehen, von psychischen Erkrankungen zu differenzieren und für Diagnostik und Behandlung nutzbar zu machen.

**Ziel:**

Ziel ist es, die theoretischen Grundlagen, Diagnostik und klinische Bedeutung des emotionalen Entwicklungsansatz für Verhaltensauffälligkeiten, psychische Störungen und Behandlungs- bzw. Interventionsplanung bei Erwachsenen mit SIE darzustellen.

**Methode:**

Der emotionale Entwicklungsansatz sowie der Zusammenhang mit herausforderndem Verhalten, psychischen Erkrankungen und entwicklungsgerechten Behandlungsmöglichkeiten bei Personen mit SIE werden dargestellt.

**Ergebnisse:**

Neben der intellektuellen Entwicklung sind bei Personen mit SIE auch sozioemotionale Fähigkeiten beeinträchtigt, wobei die Schwere von Verhaltensstörungen mit niedrigeren Entwicklungsphasen assoziiert ist. In Abhängigkeit vom emotionalen Referenzalter finden sich entwicklungstypische Verhaltensweisen, sog. Verhaltensphänomene, deren Kenntnis für die Abgrenzung von psychischen Krankheiten hilfreich ist. Bestimmte Krankheitsbilder wie z. B. Autismusspektrumstörungen, Fütter- oder Tic-Störungen treten in frühen Entwicklungsphasen häufiger auf, während andere wie z. B. dissoziative Störungen, soziale Phobien oder Persönlichkeitsstörungen erst ab einem emotionalen Referenzsalter von über 4 Jahren zu beobachten sind. Störungen wie z. B. Depression, posttraumatische Belastungsstörung und Schizophrenien treten unabhängig vom emotionalen Referenzalter in allen Entwicklungsphasen auf. Die entwicklungsgerechte Behandlungsplanung reduziert insbesondere bei Verhaltensstörungen Psychopharmaka und verbessert das Verhalten, die Stimmung und adaptiven Fähigkeiten der Personen mit SIE.

**Diskussion:**

Der emotionale Entwicklungsansatz erleichtert praktisch Tätigen die entwicklungsgerechte Kommunikation und Interaktion, unterstützt die diagnostische Abklärung und die passgenaue Therapie- und Behandlungsplanung.

## Abstract (en)

**Background:**

Individuals with intellectual disabilities (ID) show not only cognitive but also emotional developmental delays, which can be associated with challenging behavior, unnecessary psychopharmacological treatment and restricted participation. The emotional development approach provides a framework for understanding behavior associated with a certain developmental level, differentiating it from mental disorders and making it usable for diagnostics, treatment and support.

**Aim:**

The objective is to present the theoretical foundations, diagnostics and clinical relevance of emotional developmental level for behavioral problems, mental disorders and treatment or intervention planning in adults with ID.

**Method:**

The emotional development approach and current empirical studies on challenging behavior, mental disorders and developmentally appropriate treatment options in people with DID are presented.

**Results:**

In addition to intellectual development, socioemotional abilities are also impaired in people with ID, with the severity of behavioral disorders increasing in lower developmental stages. Depending on the emotional reference age, development typical behavior, so-called behavioral phenomena, can be found, knowledge of which is helpful for distinguishing them from mental illnesses. Certain clinical pictures, such as autism spectrum disorders, feeding or tic disorders occur more frequently in early developmental stages, whereas others such as dissociative disorders, social phobias or personality disorders are only observed above a developmental age of 4 years. Disorders such as depression, posttraumatic stress disorder and schizophrenia occur in all developmental stages, independent of the emotional reference age. Developmentally appropriate treatment planning reduces the use of psychotropic medication, particularly in behavioral disorders, while simultaneously improving behavior, mood and adaptive skills.

**Discussion:**

The emotional development approach enables practitioners to engage in developmentally appropriate communication and interaction, supports differential diagnostic clarification and facilitates tailored therapy and treatment planning.

In Deutschland leben ca. 1 Mio. Personen mit einer Störung der Intelligenzentwicklung (SIE, Intelligenzminderung; kognitive Beeinträchtigung; [1]). Menschen mit SIE zeigen gegenüber der Allgemeinbevölkerung vermehrt psychische und körperliche Krankheiten [2, 3] sowie eine reduzierte Lebenserwartung, wobei ein beträchtlicher Teil der vorzeitigen Todesfälle vermeidbar ist [4, 5]. Die erforderliche Diagnostik und Behandlung wird u. a. durch Barrieren in der Gesundheitsversorgung und mangelnde Fachkenntnisse auf ärztlicher Seite erschwert [6].

## Der emotionale Entwicklungsansatz

In der International Statistical Classification of Diseases and Related Health Problems 11 (ICD-11) werden Störungen der Intelligenzentwicklung (SIE) als neuromentale Entwicklungsstörungen beschrieben, die den Erwerb intellektueller, sprachlicher, motorischer und sozialer Funktionen betreffen [7,1, 8]. Neben kognitiven sind auch soziale, affektive und adaptive Fähigkeiten beeinträchtigt, die für Emotionsregulation, Erkennen emotionaler Ausdrücke und situationsangemessenes Reagieren zentral sind. Diese sozioemotionalen Fähigkeiten werden im sogenannten „sozialen Gehirn“ verarbeitet [9]. Die Entwicklung zugrunde liegender neuronaler Netzwerke verläuft schrittweise in einem Wechselspiel genetisch determinierter Prozesse und umweltbezogener Einflüsse; komplexere Funktionen bauen auf basaleren Hirnfunktionen auf [10, 11]. Die Meilensteine der neurotypischen Entwicklung spiegeln den konsekutiven Erwerb der kognitiven, emotionalen und sozialen Fähigkeiten wider [21, 22] und werden in dem Modell der emotionalen Entwicklung von Anton Došen beschrieben [12–14].

## Die emotionale Entwicklungsdiagnostik

Der emotionale Entwicklungsstand kann mit der Skala der emotionalen Entwicklung – Diagnostik 2 (SEED-2), einer Weiterentwicklung des ursprünglichen Instruments SEO (Schaal voor Emotionele Ontwikkeling) von Anton Došen, erhoben werden [15, 16]. Beide Instrumente basieren auf dem Entwicklungsmodell von Došen, das an anerkannte Positionen der Entwicklungspsychologie anschließt und sechs Entwicklungsphasen mit typischen emotionalen Referenzaltern unterscheidet:

- Adaption (1–6 Monate),
- Sozialisation (7–18 Monate),
- erste Individuation (19–36 Monate),
- Identifikation (4–7 Jahre),
- Realitätsbewusstsein (8–12 Jahre) und
- soziale Individuation (13–17 Jahre).

Die SEED‑2 ist ein standardisiertes Interview mit 240 Items, die beobachtbare Verhaltensweisen in verschiedenen Domänen beschreiben, und wurde in verschiedenen Stichproben und Sprachen psychometrisch geprüft [16–18].

## Emotionale Entwicklung und (herausforderndes) Verhalten

Verhaltensstörungen sind bei Menschen mit SIE häufig. Sie belasten Betroffene, Angehörige und Betreuungspersonen erheblich und führen oft zur Inanspruchnahme psychiatrischer Versorgung. Herausfordernde Verhaltensweisen können u. a. mit psychischen Störungen, genetisch bedingten Verhaltensphänotypen oder belastenden Umweltfaktoren zusammenhängen. Je niedriger der Entwicklungsstand, desto ausgeprägter die Verhaltensstörungen [19]. Menschen mit einem niedrigen emotionalen Entwicklungsstand verfügen nur über begrenzte Strategien zur Affektregulation; herausforderndes Verhalten ist häufig als dysfunktionale, aber entwicklungstypische Bewältigungsform zu verstehen. Die Kenntnis entwicklungstypischer Verhaltensmuster erleichtert die Abgrenzung gegenüber psychopathologischen Symptomen komorbider Störungen [20].

## Entwicklungsassoziierte Verhaltensphänomene

Das Modell von Došen unterscheidet sechs Entwicklungsphasen [24]. Im Folgenden werden Verhaltensphänomene für die Entwicklungsphasen 1 bis 4 mit den jeweiligen emotionalen Bedürfnissen, Auslösern von Problemverhalten und passenden Regulationsstrategien beschrieben, da hier besonders häufig herausfordernde Verhaltensweisen auftreten und sich in charakteristischer Weise zeigen.

### Emotionales Referenzalter 1 bis 6 Monate.

Im Vordergrund steht das Bedürfnis nach körperlichem Wohlbefinden. Schmerz, körperliches Unbehagen oder sensorische Über- bzw. Unterstimulation führen zu motorischer Unruhe, stereotypen Bewegungen, Selbstverletzung oder Rückzug. Affekte werden primär durch einfühlsame Bezugspersonen reguliert, die körperliche Nähe geben und die basalen Bedürfnisse befriedigen.

### Emotionales Referenzalter 7 bis 18 Monate.

Zentrale Bedürfnisse sind Sicherheit und Bindung. Trennungs- und Verlustsituationen, unvorhersehbare oder unstrukturierte Umgebungen provozieren impulsives, oft ungerichtet aggressives Verhalten, Distanzlosigkeit und starke Trennungsängste. Das Interesse an Peers ist gering. Stabiler (Sicht‑)Kontakt zu verlässlichen Bezugspersonen, strukturierte Abläufe und frühzeitige Deeskalation unterstützen die Affektregulation.

### Emotionales Referenzalter 19 bis 36 Monate.

Die Phase ist durch das Bedürfnis nach Autonomie geprägt. Einschränkungen der Selbstbestimmung (Verbote, erlebte Ungerechtigkeit, Machtkämpfe) führen zu trotzigem, oppositionellem und gezielt aggressivem Verhalten. Klare Strukturen, begrenzte, aber echte Wahlmöglichkeiten und ein bewusster Verzicht auf Machtkämpfe fördern die beginnende internalisierte Affektregulation.

### Emotionales Referenzalter 4 bis 7 Jahre.

Im Vordergrund steht das Bedürfnis nach Identitätsfindung und Zugehörigkeit. Ablehnung, Ausgrenzung oder beschämende Rückmeldungen in der Peergroup oder durch Bezugspersonen können zu Rückzug, gedrückter Stimmung und depressionsähnlichen Verhaltensweisen führen. Die Entwicklung von Theory of Mind, Perspektivwechsel und Scham ermöglicht zunehmend die Reflexion des eigenen Verhaltens; Anerkennung, klare Regeln und modellhaftes Lernen unterstützen die intrapersonelle Emotionsregulation.

Die auffälligen Verhaltensmuster sind vor diesem Hintergrund keine Symptome einer Krankheit, sondern entwicklungsbedingte Verhaltensphänomene. Sie werden aus den emotionalen Bedürfnissen und den begrenzten Regulationsstrategien verstehbar. Nicht medizinische Behandlungsstrategien, sondern pädagogische und psychosoziale Interventionen sind geboten. In multiprofessionellen Fallbesprechungen sollten ein entwicklungsgerechtes Verständnis und geeignete Konzepte des Umgangs im Alltag entwickelt werden. Die Kenntnis des emotionalen Entwicklungsstandes ordnet das beobachtete Verhalten ein, grenzt auffällige Verhaltensweisen von psychopathologischen Symptomen ab und gibt eine Orientierung für die Auswahl entwicklungsgerechter Umgangskonzepte.

## Emotionale Entwicklung und psychische Gesundheit

### Emotionale Entwicklung und Manifestation psychiatrischer Störungen

Der emotionale Entwicklungsstand beeinflusst nicht nur Schwere und Art von Verhaltensauffälligkeiten, sondern auch die Manifestation psychischer Erkrankungen [8, 21]. Bestimmte Störungsbilder setzen komplexe kognitiv-emotionale Fähigkeiten voraus und treten erst ab einem höheren Entwicklungsalter auf. Körperschemastörungen, dissoziative Störungen, soziale Phobien und Persönlichkeitsstörungen erfordern z. B. Integrationsleistungen und Perspektivwechsel, die etwa ab einem emotionalen Referenzalter über 4 Jahren (Phase 4) verfügbar sind. In frühen emotionalen Entwicklungsphasen (Phasen 1–3) finden sich dagegen häufiger Störungen wie Autismusspektrumstörungen, Aufmerksamkeitsdefizit‑/Hyperaktivitätsstörung (ADHS), Bindungs- und Fütterstörungen sowie Tic-Störungen. Andere Störungen wie Depression, posttraumatische Belastungsstörung und Schizophrenie können prinzipiell in allen emotionalen Entwicklungsphasen auftreten, wobei die klinische Präsentation stark vom Entwicklungsstand abhängt: Bei niedrigem Entwicklungsstand zeigen sich diese Störungen vor allem über Verhaltensänderungen und somatische Symptome, während höhere Entwicklungsphasen verbalisierte kognitive und affektive Symptome ermöglichen [8, 22, 23].

### Depression und emotionale Entwicklung

Eine Pilotstudie untersuchte den Zusammenhang von emotionalem Entwicklungsstand und Depression bei Erwachsenen mit SIE. Die Longitudinalstudie zeigte, dass depressive Episoden den emotionalen Entwicklungsstand temporär beeinflussen. Bei den untersuchten Erwachsenen mit SIE (*n* = 10) stieg das Ergebnis der emotionalen Entwicklungsdiagnostik nach Remission der Depression von 2,9 auf 3,5 Jahre (*p* = 0,034; *r* = 0,67). In der Querschnittsstudie (*n* *=* 154) zeigte sich bei Personen mit schwerer bis schwerster SIE und Depression ein niedrigerer emotionaler Entwicklungsstand als bei Personen ohne Depression (1,77 vs. 2,17 Jahre), insbesondere die soziale Interaktion und Affektregulation betreffend. Neurobiologisch sind depressive Störungen mit Beeinträchtigungen der Theory of Mind, Emotionsregulation und emotionalen Kompetenzen verbunden, also mit jenen sozioemotionalen Hirnfunktionen, die für höhere emotionale Entwicklungsphasen zentral sind [24, 25]. Diese funktionale Regression ist diagnostisch und therapeutisch relevant, da psychopathologische Symptome vor dem Hintergrund des aktuellen Funktionsniveaus einzuordnen und die Behandlung entsprechend anzupassen sind. Eine wirksame Depressionsbehandlung kann zugleich Entwicklungsspielräume eröffnen.

Die Einschätzung des emotionalen Entwicklungsstands ist sowohl im psychisch stabilen Zustand (als Baseline) als auch während akuter psychischer Krisen oder Krankheitsphasen sinnvoll, um die Behandlung und Alltagsbegleitung am aktuellen Funktionsniveau der Person auszurichten. Nach Remission kann eine erneute Einschätzung Behandlungseffekte sichtbar machen und die emotionalen Fähigkeiten im gesunden Zustand erfassen.

## Fallbeispiel

E.L., 23 Jahre mit mittelgradiger SIE im Rahmen eines Down-Syndroms, ist ein großer Ed-Sheeran-Fan (Name und identifizierende Details zum Datenschutz geändert). Sie behauptet, mit ihm verheiratet zu sein, führt intensive Gespräche mit ihrem imaginären Partner und reagiert aggressiv, wenn andere dies infrage stellen. Sie nimmt Gegenstände anderer und behauptet, diese gehörten ihr oder seien „von Ed für sie“. Mit Verdacht auf paranoid-halluzinatorische Schizophrenie wird sie psychiatrisch vorgestellt.

Die Entwicklungsdiagnostik zeigt ein emotionales Referenzalter der Phase 4. bis 7. Lebensjahr. In dieser Phase sind intensive Fantasiegeschichten entwicklungstypisch. Frau L. kann noch nicht sicher zwischen imaginierter und realer Wirklichkeit unterscheiden. Ihre Identifikation mit Ed Sheeran ist Ausdruck ihres noch ungefestigten Selbstbilds und Teil der Entwicklungsaufgabe der Ich-Bildung. Eine korrigierende Belehrung ist nicht zielführend, weil die Grenzen zwischen Fantasie und Realität noch nicht mental gefestigt sind.

Die Phantasiewelt kann gezielt pädagogisch genutzt werden („Ed würde sich doch gewiss freuen, wenn E. beim Frühstück mithilft.“), um diese in die Realität zu integrieren und das emotionale Bedürfnis nach Zugehörigkeit zu befriedigen. E.L. benötigt verlässliche emotionale Präsenz ihrer Bezugspersonen, echte Zugehörigkeitserfahrungen in der Gruppe und Unterstützung bei der Entwicklung eines realistischen Selbstbilds. Emotionale Sicherheit, pädagogisch begleitete Peer-Erfahrungen und Ich-Bildung helfen bei der Bewältigung der phasentypischen Entwicklungsaufgabe und reduzieren ggf. das Problemverhalten (Entwenden von Eigentum anderer Personen). Neuroleptika sind nicht angezeigt.

## Entwicklungslogische Behandlung und Begleitung

Die Erhebung des emotionalen Entwicklungsstands liefert Einblicke in soziale und emotionale Kompetenzen, Mentalisierungsfähigkeiten, adaptiven Fähigkeiten und Bedürfnisse einer Person. Darauf aufbauend können entwicklungsgerechte, personalisierte Interventionen abgeleitet werden [26, 27]. Personen mit einem emotionalen Referenzalter von über 4 Jahren und der damit verbundenen Fähigkeit zum Perspektivwechsel und zur Selbstreflexion profitieren eher von kognitiver Verhaltenstherapie oder mentalisierungsbasierter Therapie, während Personen mit einem emotionalen Referenzalter unter 4 Jahren eher auf körper-, bindungs- und erlebnisbasierte Verfahren ansprechen [8, 28]. Im Betreuungsalltag werden aus Unkenntnis häufig Anforderungen gestellt, die nicht entwicklungsgerecht sind und damit die Person überfordern. Fehlende personelle Kontinuität, die gerade für Personen in frühen Entwicklungsphasen zentral ist, tragen wesentlich zu herausforderndem Verhalten bei [29]. Die entwicklungsbasierte, multiprofessionelle Fallberatung ist daher ein bedeutsamer Faktor für die Passung und Wirksamkeit des gewählten Vorgehens [30].

### Reduktion von Übermedikation durch entwicklungsgerechtes Vorgehen

Eine retrospektive Studie an einer spezialisierten psychiatrischen Klinik zeigte, dass die Integration des emotionalen Entwicklungsansatzes in das Behandlungskonzept die Gesamtzahl psychotroper Medikamente und der Antipsychotikadosierung reduzierte, insbesondere bei herausfordernden Verhaltensweisen [38]. Die Berücksichtigung des emotionalen Entwicklungsstandes hilft, nicht erforderliche oder gar kontraindizierte Psychopharmaka und die damit verbundenen Risiken zu reduzieren.

### Effektivität entwicklungsbasierter Interventionen

Eine aktuelle britische Studie [39] berichtet über die Effekte von Fortbildungen des Betreuungsteams zum emotionalen Entwicklungsansatz und bindungsorientierten Betreuungsprinzipien bei 35 Personen mit SIE und herausforderndem Verhalten. Unabhängig von Alter, Geschlecht, Schweregrad der SIE und Medikation verbesserten sich unmittelbar nach dem Workshop und bis zu 6 Monate danach das Verhalten, die Stimmung und die adaptiven Fähigkeiten der Betroffenen. Das Wissen um den emotionalen Entwicklungsstand führte zu einem „Aha-Erlebnis“ im Team und zu empathischerer, strukturierterer Unterstützung.

Das Verständnis psychischer Störungen aus einer Perspektive der Entwicklungsneurobiologie und die Berücksichtigung der Bedeutung des sozialen Gehirns könnte unser Verständnis von Gesundheit und Krankheit von Menschen mit SIE verändern, die Wirksamkeit verfügbarer Behandlungen verbessern und das psychische Wohlbefinden und die soziale Teilhabe erhöhen.

## Fazit für die Praxis

- Entwicklungsdiagnostik vor Pathologisierung: Erheben Sie den emotionalen Entwicklungsstand, bevor auffälliges Verhalten als Symptom einer psychischen Erkrankung interpretiert wird.
- Differenzialdiagnostik: Unterscheiden Sie entwicklungsgerechte Bewältigungsstrategien von pathologischen Symptomen. Dies verhindert Fehlbehandlung und Übermedikation.
- Diagnostik psychischer Erkrankungen: Nutzen Sie die Kenntnis des emotionalen Entwicklungsstands für die Psychodiagnostik.
- Pharmakotherapie überprüfen: Führen Sie entwicklungslogische, pädagogische Ansätze und Alltagsbegleitung ein, wenn die Umweltanforderungen zu hoch sind, und reduzieren Sie ggf. die Psychopharmaka.
- Individualisierte Therapieauswahl: Wählen Sie Interventionen entsprechend dem emotionalen Entwicklungsstand, z. B. kognitive und mentalisierungsgestützte Verfahren ab einem emotionalen Referenzalter über 4 Jahren, körper- und bindungsbasierte Verfahren bei niedrigerem Referenzalter.

## Footnotes

1 Die noch vorläufige und unvollständige deutschsprachige Übersetzung ist als Testversion verfügbar unter https://www.bfarm.de/DE/Kodiersysteme/Klassifikationen/ICD/ICD-11/uebersetzung/_node.html. Zugriff: 11.04.2026.
